# Ingesting *Stellera chamaejasme* Significantly Impacts the Gastrointestinal Tract Bacterial Community and Diversity in Plateau Zokors (*Eospalax baileyi*)

**DOI:** 10.3390/microorganisms12112182

**Published:** 2024-10-30

**Authors:** Jialong Guo, Haijing Wang, Feng Jiang, Daoxin Liu

**Affiliations:** 1 College of Agriculture and Animal Husbandry, Qinghai University, Xining 810016, China; guojialong1011@163.com; 2 Department of Public Health, Qinghai University Medical College, Xining 810008, China; wanghj@qhu.edu.cn; 3 Qinghai Provincial Key Laboratory of Animal Ecological Genomics, Xining 810001, China; 4 Key Laboratory of Adaptation and Evolution of Plateau Biota, Northwest Institute of Plateau Biology, Chinese Academy of Sciences, Xining 810001, China

**Keywords:** plateau zokor, *Stellera chamaejasme*, gut bacteria, 16S rRNA gene

## Abstract

Intestinal bacteria are considered the “second genome” of the host, playing a crucial physiological role in assisting the host in degrading plant secondary compounds, nutrient absorption, immune regulation, and other aspects. To explore the effects of *Stellera chamaejasme* on the bacterial community of the gastrointestinal tract of plateau zokor, this study uses the 16S rRNA gene high-throughput sequencing technology, and the biodiversity and the community structure of gut bacteria in different gastrointestinal tract segments (the stomach and cecum) of plateau zokors. The results showed that at the phylum level, the dominant flora in the stomach and cecum of plateau zokors before and after ingesting *Stellera chamaejasme* were *Firmicutes* and Bacteroidetes. In plateau zokors that ingested *Stellera chamaejasme*, the relative abundance of *Firmicutes* in the stomach and cecum decreased, the relative abundance of Bacteroidetes increased, and the ratio of *Firmicutes* to Bacteroidetes decreased. After plateau zokors ingested *Stellera chamaejasme*, the ACE index demonstrated a significant reduction in the richness of the stomach bacterial community, while cecal bacterial community richness showed no significant change. *Stellera chamaejasme* exhibits significantly different effects on the bacterial communities in different segments of the gastrointestinal tract. Beta diversity analysis revealed that, after plateau zokors ingested *Stellera chamaejasme*, there were notable distinctions in the bacterial communities within both the stomach and cecum, alongside a marked reduction in the variability of the intestinal bacterial profiles across individuals. The results show that ingesting *Stellera chamaejasme* has a significant impact on the composition and structure of the gastrointestinal tract bacterial community in plateau zokors.

## 1. Introduction

Intestinal bacteria are closely associated with host health and digestion absorption, helping the host to extract energy from food [1,2]. Additionally, intestinal bacteria are instrumental in the biological degradation of plant secondary metabolites (PSMs), performing a pivotal physiological function in this process [3]. Research has found that herbivores can reduce the role of PSMs through gut bacteria in the body [4]. For example, Kohl et al. [5] found that desert lemmings (*Neotoma lepida*) fed on highly toxic *Larrea tridentata*, whose gut bacteria can enhance the host’s tolerance to phytotoxins and expand the dietary niche breadth of the host. Osawa et al. [6] and Dahlhausen et al. [7] found that Phascolarctos cinereus gut bacteria can degrade PSMs, such as Streptococcus bovis and *Lonepinella koalarum*. Currently, we understand the interaction between gut bacteria and poisonous plants, and whether gut bacteria are involved in the degradation of poisonous plants. Therefore, studying the relationship between gut bacteria and poisonous plants may help understand how the host can efficiently utilize the food resources from poisonous plants.

**Figure A1:**
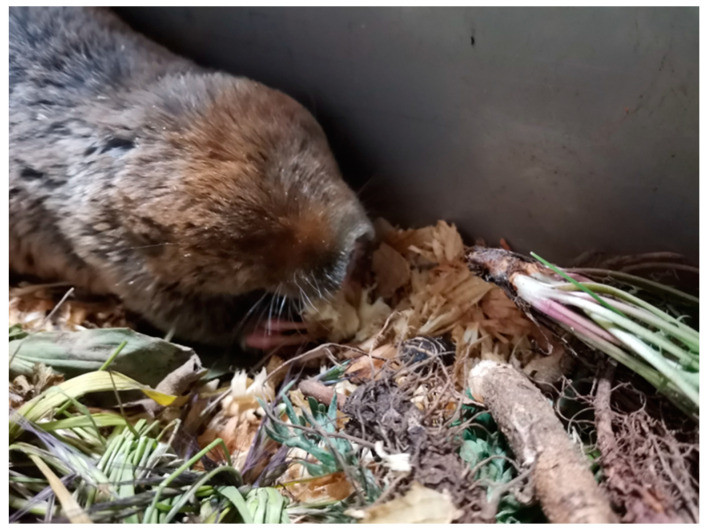
The plateau zokor actively eating *Stellera chamaejasme* even when food is abundant.

The Tibetan Plateau, the world’s largest plateau, encompasses the entirety of Tibet, as well as parts of Qinghai, northern Sichuan, western Gansu, and northwestern Yunnan [8]. *Stellera chamaejasme* L. (*Thymelaeaceae, Stellera* L.) is widely distributed in the Qinghai-Tibet Plateau of China and has a strong regeneration capacity, characterized by strong rhizomes, high soil nutrient conversion efficiency, and obvious inhibitory effects on surrounding plants, usually becoming a dominant species on grasslands [9]. *Stellera chamaejasme* is a perennial poisonous plant that thrives in sunny alpine grass slopes, lawns, and other similar environments. Its roots are characterized by a range of colors, including brown, white, yellow, and purple, while its leaves are sparse, opposite, or nearly rotund, with a long, rounded lanceolate or lanceolate shape [10,11]. The entire plant of *Stellera chamaejasme* is highly toxic. Not only does it exhibit toxic effects on bacteria and pests, but it is also extremely hazardous to humans and livestock. The ingestion of *Stellera chamaejasme* can cause severe symptoms, including diarrhea and vomiting, and in extreme cases, can be fatal [12]. The plateau zokor (*Eospalax baileyi*) is an underground rodent endemic to the Qinghai-Tibet Plateau; it mainly inhabits the alpine meadows, alpine grasslands, and shrubland in the Qinghai-Tibet Plateau [13,14]. The plateau zokor measures approximately 20 cm in length and weighs around 260 g. Due to long-term exposure to a closed, dark, high-humidity, and low-oxygen tunnel environment, its binocular vision is almost completely degraded, its forelimbs are stout, and its sense of smell and hearing is more developed [15]. Plateau zokors primarily rely on digging tunnels to find food and habitat. Their diet is basically composed of weedy plants, as they mainly feed on the roots of plants. Through digging granaries and buffet experiments, it was found that the plateau zokor exhibits tolerance and even preference for some common poisonous weeds, such as *Stellera chamaejasme*. Our previous research found that the plateau zokor will actively feed on *Stellera chamaejasme* even when food is abundant (Figure A1). The plateau zokor has admirably positioned itself as an exemplary model for investigating the degradation process of *Stellera chamaejasme* [16,17].

Therefore, this study employed 16S rRNA gene high-throughput sequencing technology in conjunction with species difference analysis to compare and analyze the composition and structure of bacterial communities within the stomach and cecum of plateau zokors both before and after the ingestion of *Stellera chamaejasme*. The objective was to investigate the impact of *Stellera chamaejasme* consumption by the plateau zokor on the intestinal bacterial community of their stomach and cecum. This research aims to provide a scientific foundation for understanding the interaction between intestinal bacteria and toxic plants.

## 2. Materials and Methods

### 2.1. Animals

Ten male plateau zokors were captured by live traps in Datong County, Qinghai Province, China (37°6′39″ N, 101°47′55″ E, elevation 2950 m). After capture, they were quickly transferred to the laboratory and reared in stainless steel cages of 40 cm× 30 cm× 25 cm. The laboratory was shaded with a shading cloth to simulate the dark environment of the underground tunnel of the plateau zokor.

### 2.2. Diet and Feeding Protocol

After the plateau zokors were captured, we initially fed them with carrots. After 20 days of feeding to adapt well, the plateau zokors were randomly divided into two groups, 5 in each group. The first group was the control group, which continued to be fed with carrots, the second group was the treatment group. For the pellets made of *Stellera chamaejasme* powder and mashed carrot, we prepared the pellets every day, and 3.92 g of *Stellera chamaejasme* powder was added per 1 kg of carrots (higher than the LD 50 in mouse) [18].

### 2.3. Sample Collection

The plateau zokor was anesthetized and killed and their stomach contents promptly extracted and immediately frozen in liquid nitrogen, with samples labeled S01 to S10. Among these, S01 to S05 constituted the experimental group (ST), whereas S06 to S10 formed the Control group (SC). Concurrently, the cecal contents were similarly removed and rapidly frozen in liquid nitrogen, with samples designated as I01 to I10. These were divided into two groups: I01 to I05 served as the experimental group (IT), and I06 to I10 were assigned to the Control group (IC). All procedures involved in the handling and care of animals were in accordance with the China Practice for the Care and Use of Laboratory Animals.

### 2.4. DNA Extraction and Sequencing

The 16S rDNA was extracted using the TruSeq^®^ DNA PCR-free sample preparation kit (Illumina, Inc., San Diego, CA, USA), and the V3-V4 region of the 16S rDNA was PCR-amplified using barcoded universal bacterial primers of 341F (5′-CCTAYGGGRBGCASCAG-3′) and 806R (5′-GGACTACNNGGGTATCTAAT-3′). PCR used TransGen AP221-02: TransStart Fastpfu DNA Polymerase; each sample had three replicates. The PCR products of the same sample were mixed and detected using 1% agarose gel electrophoresis. The PCR products were recovered by AxyPrep DNA gel recovery kit (AXYGEN, San Francisco, CA, USA) and eluted with Tris_HCl. 2% agarose gel electrophoresis. The PCR products were quantified using the Quant Fluor TM-ST blue fluorescence quantitative system (Promega, Tokyo, Japan) and then mixed in proportion to the amount of sequencing required for each sample. The library was constructed using the TruSeq TM DNA Sample Prep Kit. After the library was qualified, Illumina sequencing was performed using HiSeq 2500 for on-machine sequencing, followed by bioinformatics analysis.

### 2.5. Sequencing Data Processing

After the PE reads obtained by Illumina sequencing were split into samples, the double-ended reads were first quality-controlled and filtered according to the sequencing quality, and the overlap relationship between the double-ended reads was spliced to obtain the optimized data after quality control splicing. Operational taxonomic unit (ASV) clustering was carried out with a standard of 97% identity by uCLUST algorithm, using Vsearch v2.13.4 linux x86 64. Taxonomic assignment was searched against the SILVA123 reference database, and ASV table building was completed using the scripts of the ASV table.py in QIIME v1.9.1. Based on the representative sequence of ASV, a series of statistical and visual analyses were carried out, such as community diversity analysis, community composition analysis, and species difference analysis. ACE and Shannon indices were analyzed using mothur (version v.1.30.2) to assess community richness and diversity. R version 3.3.1 was used for the rarefaction curve, UPGMA (Unweighted Pair Group Method with Arithmetic), and PCoA (principal co-ordinates analysis). Non-metric multidimensional scaling analysis (NMDS) and similarity analysis (Anosim) were performed using R vegan software package. LEfSe analysis was performed using LEfSe 1.0 for analytical mapping.

## 3. Results

### 3.1. Data Quality

**Figure 1:**
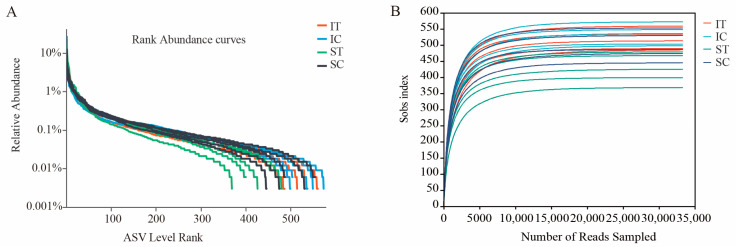
(**A**) Rank-Abundance and (**B**) Rarefaction curves of gut bacterial in plateau zokor. The number of observed ASVs gradually plateaued as the sequencing depth increased, which demonstrates that each sample had sufficient ASVs to reflect the maximum level of bacterial diversity.

In this study, a total of 1,777,901 of raw data were obtained, with an average length of 411.95 bp. After quality filtering, 9863 qualified reads were obtained, among them, the IT group had 2580 sequences, the IC group had 2657 sequences, the ST group had 2138 sequences, and the SC group had 2488 sequences. From the Rank-Abundance curve and the species dilution curve, it can be seen that with the increase in the amount of data, the curves are close to flat, indicating that the sequencing volume and sequencing depth of our data were reasonable, and a further increase in the amount of data will only find a small number of new species (Figure 1).

### 3.2. Bacterial Community Composition of Stomach and Cecum of Plateau Zokor

**Figure 2:**
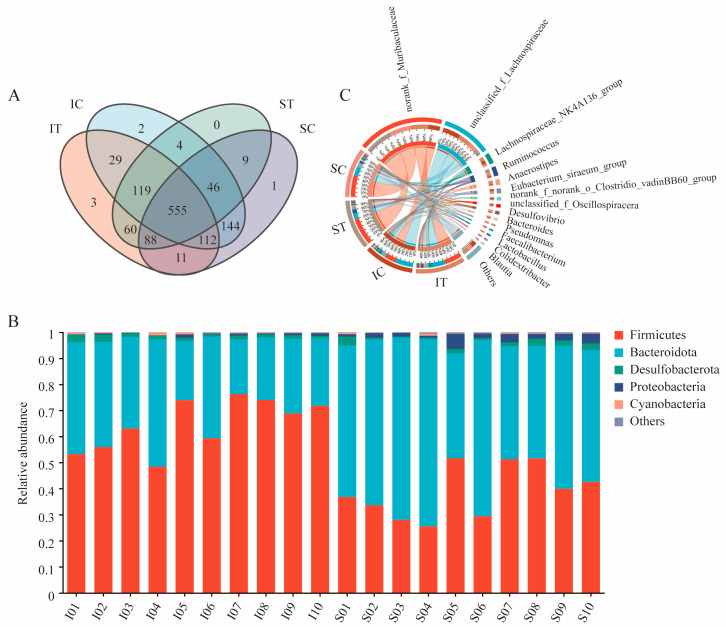
(**A**) The Venn diagram of different populations of plateau zokor at ASV level, (**B**) relative abundance histogram of gut bacteria in plateau zokor at phylum level, (**C**) Circos map of relative abundance of dominant bacteria in cecum and stomach of plateau zokor. ST and IT denote the stomach and cecum of the experimental group, respectively, whereas SC and IC represent the stomach and cecum of the control group, respectively.

In total, 3835 identified ASVs from the four groups were sorted into 10 phyla, 14 classes, 32 orders, 53 families, and 119 genera (Figure 2A).

At the phylum level (Figure 2B), before and after the plateau zokors ingested *Stellera chamaejasme*, the dominant phyla in both the stomach and cecum were the *Firmicutes* and *Bacteroidota*. The *Firmicutes* had the highest relative abundance in the stomach communities, while *Bacteroidota* were the most abundant in the cecum communities. Following the ingestion of *Stellera chamaejasme*, the relative abundance of the *Firmicutes* in the stomach decreased by 8.02%, while that of the *Bacteroidota* increased by 8.84%. In the cecum, the relative abundance of the *Firmicutes* dropped by 1.15%, and the *Bacteroidota* increased by 12.28%.

At the genus level (Figure 2C), before and after the plateau zokors ingested *Stellera chamaejasme*, the top three most abundant bacterial genera in both the ST and SC groups were the norank genus from *Muribaculaceae* family, an unclassified genus from Lachnospiraceae family, and *Ruminococcus*. The relative abundance of the first three genera in the IT group from highest to lowest was the norank genus from *Muribaculaceae* family, the unclassified genus from the Lachnospiraceae family and the *Lachnospiraceae NK4A136*. The relative abundance of the first three genera in the IC group from highest to lowest was the unclassified genus from the Lachnospiraceae family, the norank genus from the *Muribaculaceae* family and the *Lachnospiraceae NK4A136*.

### 3.3. Alpha Diversity of Gastrointestinal Tract Bacterial Community in Plateau Zokor

**Figure 3:**
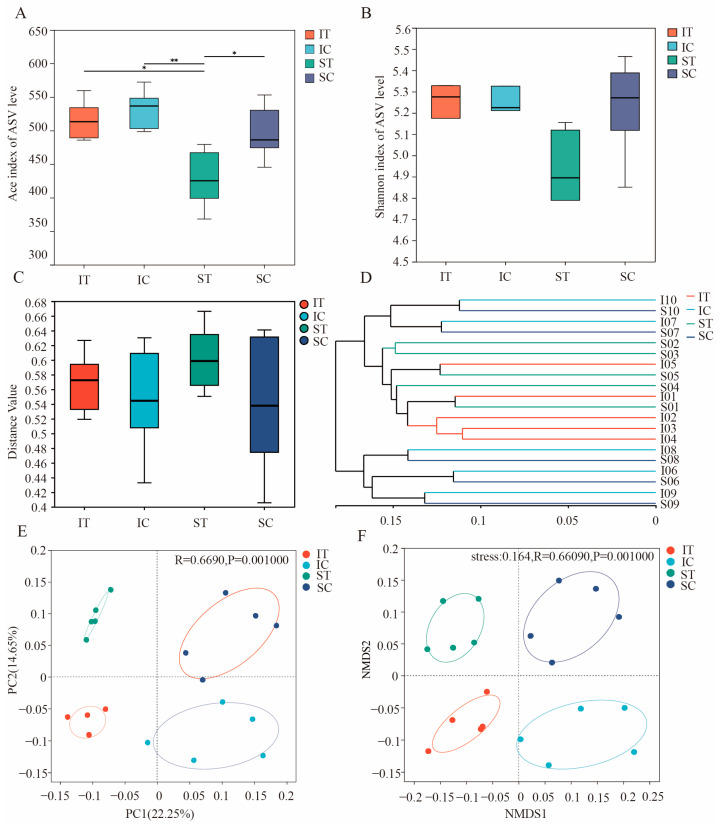
Analysis of the differences in Alpha and Beta diversity of gut bacterial communities in plateau zokor that ingested *Stellera chamaejasme* and control groups. (**A**) ACE index difference test between groups and (**B**) Shannon index difference test between groups. (**C**) Anosim Analysis Using Bray–Curtis Dissimilarity, (**D**) unweighted pair-group method with arithmetic mean (UPGMA) tree of unweighted unifrac distances. (**E**) Principal Coordinate Analysis (PCoA) using unweighted unifrac distance. (**F**) Unweighted unifrac distance-based non-metric multidimensional scaling (NMDS) analysis (0.01 < *p* ≤ 0.05 marked as *, 0.001 < *p* ≤ 0.01 marked as **). ST and IT denote the stomach and cecum of the experimental group, respectively, whereas SC and IC represent the stomach and cecum of the control group, respectively.

The Alpha diversity index (ACE and Shannon) was used for the analysis of the bacterial communities in the stomach and cecum of plateau zokor. The ACE analysis showed that after the ingestion of *Stellera chamaejasme*, there was a significant difference in richness between the ST group and the SC group in plateau zokors, while there was no significant difference in richness between the IT group and the IC group (Figure 3A). The Shannon index results indicate that the ingestion of *Stellera chamaejasme* did not significantly affect the bacterial community diversity in the stomach and cecum of plateau zokors (Figure 3B).

### 3.4. Beta Diversity of Gastrointestinal Tract Bacterial Community in Plateau Zokor

In the Beta diversity analysis, in the analyses utilizing the UPGMA clustering dendrogram (Figure 3D), Principal Coordinate Analysis (PCoA, Figure 3E), and Non-metric Multidimensional Scaling (NMDS, Figure 3F), the samples from the ST and SC groups, and the IT and IC groups, were distinctly and completely separated. Additionally, there was a notable reduction in the inter-individual variability within the gut bacterial communities of the ST and IT groups compared to the SC and IC groups. This observation was further validated by the Anosim analysis depicted (Figure 3C), which revealed a diminished inter-individual variability in the gut bacterial communities of the ST and IT groups relative to the SC and IC groups.

### 3.5. Differential Analysis of the Gut Bacterial Community in Plateau Zokors

**Figure 4:**
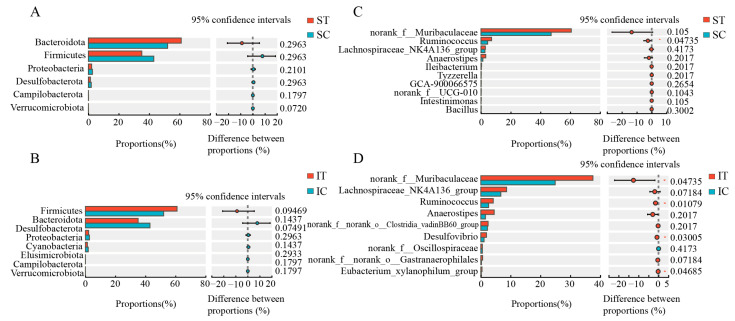
Histogram of species differences in the stomach and cecum of plateau at the phylum and genus level: (**A**) stomach species difference test histogram, (**B**) cecum species difference test histogram. (**C**) Stomach species difference test histogram, (**D**) cecum species difference test histogram. ST and IT represent the stomach and cecum of the experimental group, respectively, SC and IC represent the stomach and cecum of the control group, respectively.

At the phylum level (Figure 4A,B), before and after the plateau zokor ingested *Stellera chamaejasme*, the relative abundance of the top three dominant bacteria in the stomach was from high to low, *Bacteroidota*, *Firmicutes*, and *Proteobacteria*. The relative abundance of the first three dominant bacteria in the cecum was from high to low, *Firmicutes*, *Bacteroidota*, and Desulfobacterota. After ingesting *Stellera chamaejasme*, the relative abundance of *Firmicutes* in the stomach and cecum of plateau zokors decreased, the abundance of Bacteroidetes increased, and the ratio of *Firmicutes* to Bacteroidetes decreased, which was consistent with the analysis results of the bacterial community composition in the stomach and cecum of plateau zokors after ingesting *Stellera chamaejasme*.

At the genus level (Figure 4C,D), the three most abundant genera in the plateau zokor stomach, ranked from highest to lowest, were the norank genus from the *Muribaculaceae* family, *Ruminococcus*, followed by the *Lachnospiraceae NK4A136*. Similarly, in the cecum, the top three dominant genera, in descending order of abundance, were the norank genus from the *Muribaculaceae* family, the *Lachnospiraceae NK4A136*, and *Ruminococcus*. Notably, there was a significant increase in the relative abundance of *Ruminococcus* in the stomach following the consumption of *Stellera chamaejasme*. Furthermore, the relative abundance of the norank genus from the *Muribaculaceae* family, the *Lachnospiraceae NK4A136*, and *Ruminococcus* in the stomach and cecum increased, with a significant increase in the relative abundance of *Ruminococcus* in the stomach. In the cecum, the abundance of the norank genus from the *Muribaculaceae* family and *Ruminococcus* also showed a significant increase.

**Figure 5:**
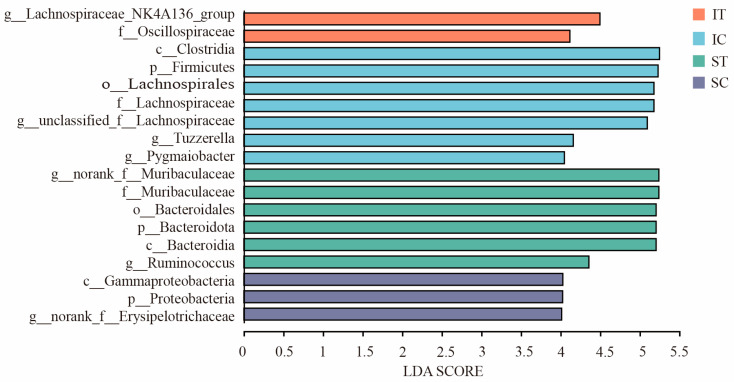
LDA score > 4 was selected as cutoff. Results of LDA effector (LEfSe) analyses. LEfSe bar graph demonstrates LDA values for different differential species in the stomach and cecum. ST and IT represent the stomach and cecum of the experimental group, respectively, SC and IC represent the stomach and cecum of the control group, respectively.

In the LEfSe analysis (Figure 5), differential biomarkers were identified with statistical significance across the IT, IC, ST, and SC groups, with 2, 7, 6, and 3 basic biomarkers. For the IT group, the biomarkers included the *Lachnospiraceae NK4A136* group and Oscillospiraceae. In the IC group, significant biomarkers were observed for Clostridia, *Firmicutes*, *Lachnospirales*, Lachnospiraceae, the unclassified genus from the Lachnospiraceae family, *Tuzzerella*, and *Pygmaiobacter*. The ST group was characterized by the norank genus from the *Muribaculacea* family, *Muribaculaceae*, *Bacteroidales*, *Bacteroidota*, *Bacteroidia*, and *Ruminococcus.* For the SC group, the biomarkers were *Gammaproteobacteria*, *Proteobacteria*, and the norank genus from the *Erysipelotrichaceae* family. For the SC group, the biomarkers were *Gammaproteobacteria*, *Proteobacteria*, and the norank genus from the *Erysipelotrichaceae* family. At the phylum level, bacterial communities with greater abundance in the IT and IC groups were predominantly *Firmicutes*, while the ST group was enriched in both Bacteroidetes and *Firmicutes*, and the SC group was concentrated in *Proteobacteria* and *Firmicutes*. At the genus level, the biomarker for the IT group was the *Lachnospiraceae NK4A136*, whereas the IC group was marked by *Tuzzerella*, *Pygmaiobacter*, and the unclassified genus from the Lachnospiraceae family; in the ST group, *Ruminococcus* and the norank genus from the *Muribaculaceae* family were the prominent biomarkers, and in the SC, group was the norank genus from the *Erysipelotrichaceae* family.

### 3.6. PICRUSt2 Function Prediction

**Figure 6:**
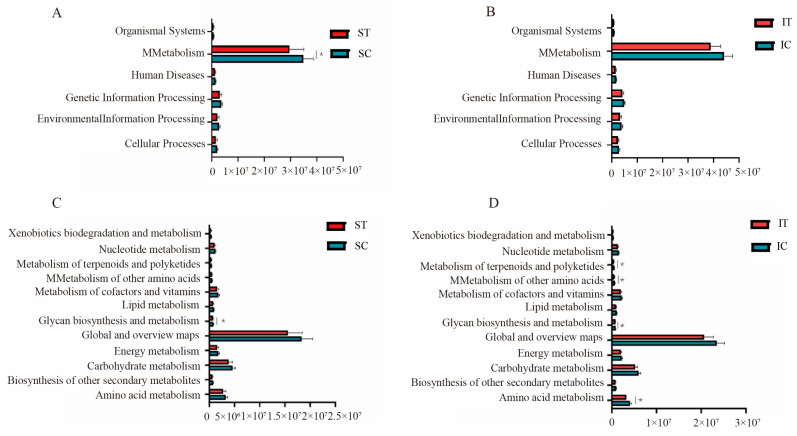
Relative abundance of predicted function for specific KEGG modules (level 1 and level 2). (**A**) Relative abundance map of stomach function prediction at level 1. (**B**) The relative abundance map of cecum function prediction at level 1. (**C**) Relative abundance map of stomach function prediction at level 2. (**D**) The relative abundance map of cecum function prediction at level 2. (0.01 < p ≤ 0.05 marked as *). ST and IT represent the stomach and cecum of the experimental group, respectively, SC and IC represent the stomach and cecum of the control group, respectively.

The KEGG (Kyoto Encyclopedia of Genes and Genomes) database was compared (Figure 6). In the six biological functional metabolic pathways of level 1, after the plateau zokor ingested *Stellera chamaejasme*, the relative abundance of stomach metabolism was significantly decreased, whereas the six biological functional metabolic pathways in the cecum did not exhibit significant changes. In level 2, after the plateau zokor ingested *Stellera chamaejasme*, significant differences were observed in the stomach in relation to gycan biosynthesis and metabolism pathways. There were significant differences in metabolism of terpenoids and polyketides, metabolism of other amino acids, glycan biosynthesis and metabolism, and amino acid metabolism in cecum.

## 4. Discussion

### 4.1. Stellera chamaejasme Alters the Gastrointestinal Bacterial Community of Plateau Zokors

The abundance and composition of gut bacterial communities have been proven to have a significant impact on the host’s nutrition and metabolism, as well as on immune function. Gut bacteria may play a crucial role in assisting herbivorous animals in the degradation of poisonous plants [19,20]. This study investigates the effects of plateau zokors ingesting *Stellera chamaejasme* on the bacterial communities in their stomachs and cecum. The results showed that at the phylum level, the dominant flora in the stomach and cecum of plateau zokors before and after the ingestion of *Stellera chamaejasme* were *Firmicutes* and Bacteroidetes. The *Firmicutes* in the intestine possess robust degradation capabilities, which enable the breakdown of dietary fiber to facilitate the degradation of polysaccharides, ultimately benefiting the host by enhancing energy extraction from food. Bacteroidetes, on the other hand, play a crucial role in degrading carbohydrates and proteins, thereby improving the efficiency of nutrient utilization and facilitating the digestion and absorption of nutrients for the host [21,22]. In studies of gut bacterial communities in species like the Gansu Zokor and New Zealand white rabbits, which are herbivorous animals, *Firmicutes* and Bacteroidetes represent the two most prevalent phyla within the gut’s bacterial community, constituting the largest proportion of bacteria present [23,24]. Ingesting *Stellera chamaejasme* by the plateau zokor led to a reduction in the ratio of *Firmicutes* to Bacteroidetes. The study revealed that this trend was also evident in the gut bacteria of wild mice, and a lower *Firmicutes* to Bacteroidetes ratio is widely regarded as conducive to enhancing the absorption of dietary fat and energy [25,26].

At the genus level, subsequent to the plateau zokor ingesting *Stellera chamaejasme*, the relative abundance of the norank genus from the *Muribaculaceae* family, the *Lachnospiraceae NK4A136*, and *Ruminococcus* in the stomach and cecum increased, with a significant increase in the relative abundance of *Ruminococcus* in the stomach. In the cecum, the abundance of the norank genus from the *Muribaculaceae* family and *Ruminococcus* also showed a significant increase. *Ruminococcus* belongs to the *Firmicutes* phylum and aids in the degradation of indigestible substances such as cellulose and hemicellulose in the intestines, promoting the absorption of sugars, providing the host with the necessary nutrients [27]. In addition, *Ruminococcus* contributes to the formation of butyrate, thereby supporting the maintenance of a healthy intestinal barrier [28]. Amato et al. [29] found that during periods of decreased energy intake, there is a marked surge in the abundance of *Ruminococcus* within the gut microbiome of monkeys (*Alouatta pigra*). Furthermore, *Ruminococcus* has the ability to generate anti-inflammatory short-chain fatty acids (SCFAs), which are of paramount importance in modulating intestinal inflammation [30]. Choy et al. found that the supplementation of phenolic compounds in sow diets is associated with a marked increase in the relative abundance of *Ruminococcaceae* bacteria. The norank genus from the *Muribaculaceae* family represents a dominant genus within the Bacteroidetes order, which is frequently encountered within the gastrointestinal tract [31]. Esteemed as a pivotal bacterium in the regulation of inflammation, the norank genus from the *Muribaculaceae* family not only bolsters the barrier function of the gastrointestinal mucosal layer but also exhibits a negative correlation with the individual’s inflammatory state [32,33]. Furthermore, the norank genus from the *Muribaculaceae* family plays a crucial role in the breakdown of cellulose and in the efficient acquisition of energy [34]. The *Lachnospiraceae NK4A136* is a commensal bacterium that is favorable for intestinal health, exhibiting pronounced anti-inflammatory effects and performing a critical function in the maintenance of intestinal homeostasis [35]. The administration of the *Lachnospiraceae NK4A136* was found to ameliorate hyperlipidemia and insulin resistance in obese mice [36]. The study reveals that secondary plant compounds are capable of promoting both the wellbeing of animal intestines and the entire organism. This is achieved through the augmentation of beneficial bacterial populations within the intestines and the suppression of the establishment of harmful bacteria in the gastrointestinal tract [37]. Phenols and flavonoids, alongside other secondary plant compounds, contribute positively to anti-inflammatory responses, antimicrobial functions, and the maintenance of gastrointestinal health [38,39]. KEGG pathway analysis revealed significant alterations in the enrichment of functional genes of intestinal bacteria in the stomach and cecum. After the plateau zokor consumed *Stellera chamaejasme*, some KEGG level 1 and 2 functional categories showed significant differences between groups, but the relative abundance of these categories and all other detected categories decreased. It is inferred that plateau zokor may slow down the absorption of the *Stellera chamaejasme* toxin by reducing metabolism function. After ingesting *Stellera chamaejasme*, the stomach and cecum experience a shift in bacterial community structure. This transformation may contribute to the suppression of inflammation by boosting the prevalence of specific bacterial species. It is plausible that this is one of the mechanisms by which the plateau zokors can efficiently metabolize *Stellera chamaejasme*. Additionally, *Stellera chamaejasme* could be pivotal in safeguarding plateau zokors against gastrointestinal inflammation.

### 4.2. Stellera chamaejasme Altered the Diversity of the Gastrointestinal Bacteria in Plateau Zokors

In the alpha diversity analysis, upon the ingestion of *Stellera chamaejasme* by plateau zokors, the ACE index indicated a pronounced decrease in the diversity of bacteria within the stomach community, whereas the cecal bacterial diversity remained largely unchanged. *Stellera chamaejasme* exerted significantly varied influences on the bacterial populations across different segments of the gastrointestinal tract. There is a notable discrepancy in the composition and diversity of bacterial communities among various segments of the animal’s gastrointestinal tract [40]. Due to the low pH value of the stomach, it is difficult to ensure the growth of bacteria and maintain a richer diversity [41]. The cecum, serving as the primary fermentation organ in plateau zokors and having a neutral pH value conducive to bacterial growth, allows for the maintenance of relatively high levels of bacterial diversity and richness [42]. The stomach serves as the temporary storage site for food in animals, where it undergoes the preliminary stages of digestion and absorption. Cai et al. [43] found that the plateau zokor did not achieve food digestion through enlargement of the gastrointestinal tract, but rather through the evolution of a complex stomach and developed gastrointestinal tract. This study revealed that ingesting *Stellera chamaejasme* exerted a more significant influence on the bacterial community within the plateau zokor’s stomach compared to the cecum. This leads to the hypothesis that the toxins present in *Stellera chamaejasme* are primarily metabolized within the zokor’s stomach. Nonetheless, the validity of this stomach-centric detoxification strategy for *Stellera chamaejasme* in plateau zokors awaits verification through subsequent research investigations.

In the Beta diversity analysis, after the plateau zokors have ingested *Stellera chamaejasme*, the UPGMA clustering tree, PCoA, and NMDS analyses have shown the bacterial communities in the stomach and cecum both exhibit significant differences. This research indicates that secondary plant compounds are capable of engaging in a multitude of biological transformation processes within animal organisms, thereby modifying the ecological niche of gut bacteria. This alteration subsequently influences the diversity, abundance, and equitability of the bacterial community structure [19]. Following the ingestion of toxic plants, animals such as koalas, black howler monkeys, and plateau pikas undergo a noticeable shift in the composition of their gut bacterial communities [6,7,19]. Clearly, the ingested *Stellera chamaejasme* by plateau zokors leads to a substantial alteration in the architecture of their intestinal bacterial communities, indicating its capacity to potentially reconstitute the composition and structure of the zokor’s gut bacterial.

### 4.3. Stellera chamaejasme Induces Convergent Changes in the Gut Bacterial Communities Among Plateau Zokor Individuals

The composition of the gastro gut bacterial flora is influenced by numerous factors, including sex, diet, and geographical distance, among others. Food is an important factor that significantly impacts the alteration of gut bacterial communities in rodents [44,45]. In previous studies, Liu et al. [46] found that consistent food did not converge the gut bacteria of the plateau zokor. Contrarily, in this study, the UPGMA clustering tree, PCoA, NMDS, and Anosim results suggest that after the ingestion of *Stellera chamaejasme*, the differences in the intestinal bacterial communities among individual plateau zokors had decreased. Secondary metabolites from plants typically contain active components that exhibit antimicrobial, insecticidal, and other bioactive properties [47]. The study reveals that *Stellera chamaejasme* possesses strong antiproliferative activity against tumor cells, and it also exhibits antimicrobial, antiviral, and insecticidal properties [12]. Guessing that after plateau zokors ingested *Stellera chamaejasme*, the antibacterial activity may inhibit the growth and reproduction of certain intestinal bacteria, which in turn alters the species diversity and relative abundance of gut bacteria, leading to a reduction in the inter-individual differences within the gut bacterial communities of the plateau zokors. At present, the mechanism of interaction between toxic plants and gut bacteria remains unclear. Therefore, further research is needed to investigate the bidirectional effect of mutual changes between gut bacteria and toxic plants.

## 5. Conclusions

This research, which entailed analyzing and contrasting the shifts in bacterial communities within the stomach and cecum of plateau zokors, both before and after ingesting *Stellera chamaejasme*, indicated that in both the stomach and cecum, the dominant bacterial phyla remained the *Firmicutes* and Bacteroidetes. During the alpha diversity analysis, it was observed that the ingestion of *Stellera chamaejasme* by the plateau zokors was more impactful on the richness of the stomach’s bacterial community compared to that of the cecum. Beta diversity analysis reveals that, after plateau zokors ingested *Stellera chamaejasme*, there were notable distinctions in the bacterial communities within both the stomach and cecum, alongside a marked reduction in the variability of the intestinal bacterial profiles across individuals. The results show that the ingestion of *Stellera chamaejasme* has a significant impact on the composition and structure of the gastrointestinal tract bacterial community in plateau zokors. It is hypothesized that the intestinal microorganisms of the plateau zokor may play a role in degrading the toxins present in *Stellera chamaejasme*, although further research is necessary to elucidate the specific mechanisms involved.

## Data Availability

Sequence data are available from Sequence Read Archive (SRA) BioProject PRJNA1170538.
